# RETRACTION: Macrophages of the M1 and M2 Types Play a Role in Keloids Pathogenesis

**DOI:** 10.1111/iwj.70664

**Published:** 2025-04-23

## Abstract

**RETRACTION:** SeoudyW.M. , El DienS.M. Mohy , ReheemT.A.A. , ElfangaryM.M. , and ErfanM.A. , “Macrophages of the M1 and M2 Types Play a Role in Keloids Pathogenesis,” International Wound Journal 20, no. 1 (2023): 38–45, 10.1111/iwj.13834.35604036 PMC9797913

The above article, published online on 23 May 2022, in Wiley Online Library (http://onlinelibrary.wiley.com/), has been retracted by agreement between the journal Editor in Chief, Professor Keith Harding; and John Wiley & Sons Ltd. Following an investigation by the publisher, all parties have concluded that this article was accepted solely on the basis of a compromised peer review process. The editors have therefore decided to retract the article. The authors did not respond to our notice regarding the retraction.

**RETRACTION:** W.M. Seoudy , S.M. Mohy El Dien , T.A.A. Reheem , M.M. Elfangary , and M.A. Erfan , “Macrophages of the M1 and M2 Types Play a Role in Keloids Pathogenesis,” International Wound Journal 20, no. 1 (2023): 38–45, 10.1111/iwj.13834.35604036 PMC9797913

The above article, published online on 23 May 2022, in Wiley Online Library (http://onlinelibrary.wiley.com/), has been retracted by agreement between the journal Editor in Chief, Professor Keith Harding; and John Wiley & Sons Ltd. Following an investigation by the publisher, all parties have concluded that this article was accepted solely on the basis of a compromised peer review process. The editors have therefore decided to retract the article. The authors did not respond to our notice regarding the retraction.

